# c-Myc Represses Tumor-Suppressive microRNAs, let-7a, miR-16 and miR-29b, and Induces Cyclin D2-Mediated Cell Proliferation in Ewing’s Sarcoma Cell Line

**DOI:** 10.1371/journal.pone.0138560

**Published:** 2015-09-22

**Authors:** Masanori Kawano, Kazuhiro Tanaka, Ichiro Itonaga, Tatsuya Iwasaki, Hiroshi Tsumura

**Affiliations:** Department of Orthopaedic Surgery, Faculty of Medicine, Oita University, Oita, Japan; University of Illinois at Chicago, UNITED STATES

## Abstract

Myc oncogenic transcription factor is known to inhibit tumor suppressive microRNAs (miRNAs), resulting in greater expression of their target protein related to cell cycle, invasion or anti-apoptotic factors in human cancer cells. To explore possible oncogenic factors in Ewing’s sarcoma (ES), we conducted microarray-based approach to profile the changes in the expression of miRNAs and its downstream mRNAs in five ES cell lines and human mesenchymal stem cells (hMSCs). Three miRNAs, let-7a, miR-16 and miR-29b were significantly down-regulated, whereas c-Myc and cyclin D2 (CCND2) were significantly up-regulated in all tested ES cells compared with hMSCs. To verify that let-7a, miR-16 and miR-29b were the targets of c-Myc in ES cell lines, we transfected siRNA against c-Myc and confirmed the coordinate up-regulation of let-7a, miR-16 and miR-29b through the repression of c-Myc. The ES cells transfected with c-Myc-siRNA and let-7a, miR-16 and miR-29b exhibited the inhibition of the cell cycle progression. The increased expression of let-7a, miR-16 and miR-29b resulted in the reduction of CCND2 protein expression. We also demonstrated that c-Myc-siRNA treatment of ES cells was associated with the decreased expression of CCND2 as a down-stream of three miRNAs. Furthermore, the introduction of let-7a, miR-16 and miR-29b in ES cells could inhibit the c-Myc-mediated up-regulation of CCND2 resulted in the prevention of cell cycle progression. In addition, the transfection of let-7a, miR-16 and miR-29b in ES cells suppressed tumor growth ex vivo treatment. These findings suggests that the up-regulation of c-Myc inhibited the expression of let-7a, miR-16 and miR-29b subsequently induced CCND2 expression in ES cells. The present study might identify a novel oncogenic axis that c-Myc regulates the expression of CCND2 via let-7a, miR-16 and miR-29b, leading to the development new therapeutic targets for ES.

## Introduction

Ewing’ sarcoma (ES) is the second most common bone cancer, most often occurring in children, adolescents, and young adults. ES is considered as the high-grade malignancy and quickly metastasize to the bone marrow, lung, and other tissues [1]. Unfortunately, approximately 30% of ES patients have metastases at presentation. The patients with metastatic ES have drastically worse outcomes since intensive systemic chemotherapy failed to improve survival of the patients [2].

MYC oncogene, which is amplified in many human cancers including ES, encodes a transcription factor c-Myc, and affects the cellular behaviors such as cell growth, metabolism, survival and chromosomal translocations in human cancers [3]. c-Myc controls the cell cycle by operating the levels of several regulators of progression through G1 such as cyclins and CDKs. c-Myc also controls the production of many non-coding RNAs, including micro-RNAs (miRNAs), and these miRNAs are likely to contribute substantially to the biologic and pathologic functions of c-Myc [4].

miRNAs are single-stranded non-coding single stranded RNAs (18–24 nucleotides) that are capable of inhibiting gene expression at the post-transcriptional level referred to as RNA interference (RNAi) [5]. miRNAs exhibit sequence specific interaction with the 3’-untranslated region (UTR) of cognate mRNA targets, causing degradation of mRNAs and suppression of translation [6]. miRNAs have identified as key regulators of multiple physiological and pathological processes, including cell proliferation, apoptosis, and cancer [7,8]. In the past decade, emerging evidences have demonstrated a diverse function of miRNAs in the establishment and progression of human tumors. miRNAs can either regulate or act as oncogenes [9,10] or tumor suppressor genes [11] at the post-transcriptional level.

D-type cyclins are an important group of highly conserved cell cycle regulators. A family member of D-type cyclins, cyclin D2 (CCND2), is the key player in cell cycle progression from the G1 phase to S phase [12]. It has been reported that CCND2 was overexpressed due to either amplification of CCND2 genes or aberrant mitogenic signaling in several types of sarcoma [13, 14]. Although several miRNAs have been found to target CCND2, including let-7a [15], miR-16 [16] and miR-29b [17], the correlation of CCND2 expression and miRNAs in ES cells has been unknown.

In the present study, we analyzed genome wide array expression of both miRNAs and mRNAs in five human ES cell lines and human mesenchymal stem cells (hMSCs). The results herein indicated that the expressions of let-7a, miR-16 and miR-29b were repressed whereas those of c-Myc and CCND2 were increased in all five ES cell lines compared with hMSCs. Based on the inverse correlation between let-7a/miR-16/miR-29b and c-Myc and CCND2 expression, we hypothezed that down regulation of c-Myc would restore the expression of tumor-suppressive miRNAs, let-7a, miR-16 and miR-29b, subsequently down-regulate CCND2 in ES cell lines. The aim of our study is to provide novel insight into the mechanism, by which tumor suppressive miRNAs are reduced via c-Myc resulting in up-regulates CCND2, as the potential therapeutic target for ES.

## Material and Methods

### Ethics statement

The animal experimental protocol was approved by the Ethics Review Committee for Animal Experimentation of Oita University, and all mice used in this study were anesthetized with ketamine/xylazine or isoflurane/oxygen for experiments and euthanized with cervical dislocation under anesthesia. All efforts were made to minimize suffering.

### Mice

BALB/c nu/nu mice, (n = 25, 6 week old,), were acquired from the Kyodo Laboratory (Tosu, Japan). After quarantine, all mice were kept in a pathogen free environment on a standard 12hr-day/12hr-night cycle and were fed a standard sterilized pellet diet and water *ad libitum*. All mice were sacrificed 6 weeks after the cell inoculation or when the subcutaneous tumors grew to 2000 mm^3^. They were continuously monitored during daytime from Monday to Friday, and twice daily during daytime on Saturdays, Sundays, and holidays for signs of poor health.

### Cell lines

The human ES cell lines, SKES1, RDES, SKNMC and SCCH were obtained from JCRB Cell Bank (Tokyo, Japan), and WE68 was kindly provided Dr. Frans van Valen (Westfalische-Wilhelms University, Munster, Germany). Human mesenchymal stem cells (hMSCs) were purchased from TaKaRa Biotechnology (Japan). Each line was authenticatedas to genotype and phenotype by the source company. RDES and SKNMC cells were cultured in Dulbecco’s modified eagle medium (DMEM) high glucose medium (Invitrogen, NY) with 10% FBS and 1% penicilium and streptomycin. SKES1 cells were cultured in RPMI 1640 (Invitrogen, NY) supplemented with 10% FBS. SCCH cells were grown in minimal essential medium (MEM) supplemented with 10% fetal bovine serum (FBS; Invitrogen, NY) and 0.1 mmol/L nonessential amino acids (NEAA). hMSC were cultured Mesenchymal Stem Cell Basal Medium, Chemically Defined (MSCBM-CD) with MSCGM-CD SingleQuats (TaKaRa Bio). Cells were maintained at 37°C incubator supplied with 5% CO_2_ and passaged every 2 to 3 days.

### RNA isolation

mRNAs were prepared from the triplicated cell cultures using RNeasy kit (Qiagen, Valencia, CA) according to the manufacturer’s instruction. The RNA quality was ensured, before labeling, using RNA 6000 Nano kit and Bioanalyzer 2100 (Agilent, Santa Clara, CA). miRNAs were prepared from triplicate cell cultures using the miRNeasy Mini kit (Qiagen) according to the manufacturer’s instruction.

### Genome-wide miRNA expression microarray

GeneChip miRNA 3.0 array (Affymetrix, Santa Clara, CA) was used for miRNA expression profiling in five ES cell lines and hMSC. One μg of small RNA including miRNA from each sample was labeled with biotin using the FlashTag Biotin HSR Kit (Genisphere, Hatfield, PA). Array hybridization, washing and scanning of the slides were carried out according to the manufacturer’s recommendations. The data were extracted from the images, quantile-normalized, summarized (median polish), and log2-transformed with miRNA QC software (Affymetrix). GeneSpring GX 11.0 (Agilent) was used to analyze the array results. Analysis of variance was used to determine those probe sets significantly different between the two groups. The gene list was filtered with a fold-change cutoff of 2, resulting in the output of list with genes that have significant differential expression at 2-fold or more differences. Pathway analysis was performed using KEGG PATHWAY Database (http://www.genome.jp/kegg/pathway.html).

### Analysis of mRNA expression by cDNA arrays

GeneChip Genome HG U133 Plus 2.0 Array (Affymetrix) was used for mRNA expression profiling in five ES cell lines and hMSC. Biotinylated cRNA was synthesized from total RNA using the 3’ IVT Express Kit (Affymetrix) according to the manufacturer’s protocols. In brief, double stranded cDNA was generated by the reverse transcription from 1 ng of total RNA using an oligo (dT) primer bearing a T7 promotor. The double-strand cDNA was used as a template for in vitro transcription to generate biotin-labeled cRNA. After fragmentation, 12.5 μg of cRNA were hybridized to GeneChip array for 16 hr. The arrays were washed and stained using GeneChip Fluidics Station 450 (Affymetrix) and then scanned with the GeneChip Scanner 3000 (Affymetrix). The entire experiment was performed twice. Array hybridization, washing, and scanning of the slides were carried out according to the manufacturer’s protocols. The microarray numerical values were analyzed using the GeneSpring GX 11.0 software, according to the RAM16 Algorithm [18]: quantile normalization, filter by flags (detected), filter by expression on the normalized data (20.0–100.0th percentile). Analysis of variance was used to determine those probe sets significantly different between the two groups. The gene list was filtered with a fold-change cutoff of 2, resulting in the output of list with genes that have significant differential expression at 2-fold or more differences.

### Mature miRNA transfection

One day prior to the transfection, cells were seeded onto 6 well plates (5x10^4^ cells/well) and incubated with the complete medium without antibiotics (2 ml/well). The transfection of let-7a-2-3p mimic (Accession; MIMAT0010195), miR-16-2-3p mimic (Accession; MIMAT0004518) and miR-29b-1-5p (Accession; MIMAT0004514) mimic or negative control (NC) miRNA (Invitrogen) was performed using Lipofectamine 2000 reagent (Invitrogen) in antibiotics-free OptiMEM (Invitrogen) according to the manufacturer's instructions. After 48 h incubation following the transfection, the cells were harvested and processed for further analysis.

### Prediction of binding site and mature miRNA transfection

Among the predicted target genes of let-7a, miR-16 and miR-29b in the TargetScan (http://www.targetscan.org/), DIANA (http://diana.cslab.ece.ntua.gr/microT/), and PicTar (http://pictar.mdc-berlin.de/) databases, we found that CCND2 was one of the top candidates (S1 Fig). One day prior to the transfection, cells were seeded onto 6 well plates (5x10^4^ cells/well) and incubated with the complete medium without antibiotics (2 ml/well). Actinomycin D (10 μg/ml, Sigma-Aldrich) was used to inhibit nascent RNA synthesis. The transfection of let-7a mimic, let-7a mutant, miR-16 mimic, miR-16 mutant, miR-29b mimic, miR-29b mutant and negative control mRNAs (Control-miR) (Invitrogen) was performed using Lipofectamine 2000 reagent (Invitrogen) in antibiotics-free OptiMEM (Invitrogen).

### RNA extraction, cDNA synthesis, and quantitative real time PCR

Total RNA was extracted from prepared tumor samples with the TRizol reagent (Invitrogen) and cDNA was synthesized according to the manufacturer's protocol (Roche). Quantitative real-time PCR (qRT-PCR) was performed using a Light Cycler 480 Probe Master System (Roche), and PCR-specific amplification was conducted in the LightCycler® Nano (Roche). The relative expression of genes (CCND2 and GAPDH) was calculated with the 2- (ΔΔCt) method. The primers used are listed here (qRT-PCR; CCND2-forward 5'- AAGAATTCCTCCTCAATAGCCTGCAGCAGTA -3', CCND2-reverse 5'- GCGGGATATCGACCTGTGAGAATTCGAT -3';GAPDH-forward 5'-CCTCTATGCCAACACAGTGC-3', GAPDH-reverse 5'-GTACTCCTGCTTGCTGATCC-3'. PCR was performed under the following conditions: 50°C for 2 min, 95°C for 10 min, followed by 50 cycles at 95°C for 15 s, and 60°C for 1 min. Each sample was run in triplicate.

### Knockdown of c-Myc and CCND2 expression using siRNA

siRNA oligonucleotides targeting c-Myc (Entrez Gene; 4609, Accession; NM_002467) and CCND2 (Entrez Gene; 894, Accession; NM_001759) mRNA was purchased from Ambion (Tokyo, Japan) and MISSION siRNA Universal Negative Control was purchased from Sigma Aldrich (Osaka, Japan). The siRNAs were transfected into SKES1 and RDES cells using Lipofectamine 2000 reagent according to the manufacturer's instructions. The cell lines were harvested 48 h after the transfection, and subjected to various analyses. The experiment was repeated three times.

### Cell proliferation assay

The cells were plated in 6-well plates (5×10^4^ cells per well), and were transfected with or without let-7a-2-3p, miR-16-2-3p and miR-29b-1-5p mimic, negative control miRNA, or CCND2 siRNA and MISSION siRNA Universal Negative Control. Then the cells were incubated in antibiotic-free OptiMEM. After 48 h of the cultivation, the cells were counted using TC10 Automated Cell Counter (BioRad).

### Western blot

Total cellular protein (15 μg) was resolved on a precast 10% Tris–HCl Criterion 10-well gel (Biorad) at 200 V (300 mAmp) for 30 min. The gel was wet-transferred to a PVDF membrane for 1 h, and blocked with PBST containing 5% instant dry non-fat milk for 30 min at room temperature. Antibodies against c-Myc protein (#9402), CCND2 (#2924) were obtained from Cell Signaling and β-Actin (ab16039) proteins were obtained from Abcam (Cambridge, UK). Immunocomplexes were visualized with horseradish peroxidase-conjugated anti-rabbit immunoglobulin G antibodies (GE Healthcare, Tokyo, Japan), developed the blots using ECL Plus system (GE Healthcare) with a ChemiDoc camera (ImageQuant LAS 4000mini; GE Healthcare). The quantification of western blot signals was performed by the densitometry with ImageQuant TL software (GE Healthcare). All western blot experiments were repeated at least three times.

### Cell cycle analysis

For cell cycle analysis, cells were stained with propidium iodide using Cycletest Plus DNA Reagent Kit (BD Biosciences) following the manufacturer’s protocol, and the cell cycle distribution was analyzed by FACSVerse flowcytometer (BD Biosciences). The percentages of cells in G0⁄G1, S, and G2⁄M phases were counted and compared. The experiments were carried out in triplicate.

### Apoptosis assay

The quantification of cell death was determined by fluorescence activated cell sorting (FACS) using Annexin V-FITC apoptosis detection kit (BD Bioscience) according to the manufacturer’s instructions. Briefly, 1x10^6^ SKES1 cells were seeded and incubated for 24 h, then let-7a-2-3p, miR-16-2-3p and miR-29b-1-5p mimic or siRNA for CCND2 was added to the cells followed by incubation for 48 h. The cells were washed with PBS, suspended in annexin V binding buffer, then added to an annexin V-FITC solution and propidium iodide (PI) for 20 min at room temperature. The samples were analyzed by FACSVerse using FACSuite analysis software (BD Bioscience). As a positive control for apoptosis, SKES1 cells treated with doxorubicin at 40 mg/ml for 20 h were used.

### Ex vivo tumor bearing nude mice models

The experimental tumor bearing mice model was established by injection of SKES1 cells (1 x 10^6^) transfected with let-7a-2-3p, miR-16-2-3p and miR-29b-1-5p miRNA suspended in 100 μl of normal saline in the gluteal region of mice. Five groups were generated: (1) untreated control (n = 5); (2) transfected with negative control-miRNA (n = 5); (3) transfected with let-7a miRNA mimic (n = 5); (4) transfected with miR-16 miRNA mimic (n = 5); (5) transfected with miR-29b miRNA mimic (n = 5). Tumor size was measured in two perpendicular dimensions parallel with the surface and the depth of the tumor in mice using a caliper. The tumor volume was estimated using the formula V = (Length × Width^2^)/2.

### Statistical analysis

Statistical analysis was carried out using SPSS 16.0 for Windows (SPSS, IL). Two-tailed Student’s t-test was used for analysis of continuous variables. P < 0.05 and 0.01 were considered to be statistically significant. We determined differences more 3 groups using a nonrepeated measures analysis of variance (ANOVA) and Scheffe test. Results were expressed as the mean ± standard deviation, and p < 0.05 and p < 0.01 was considered as statistically significant.

## Result

### Down regulation of let-7a, miR-16, and miR-29b expression in ES cell lines

**Fig 1:**
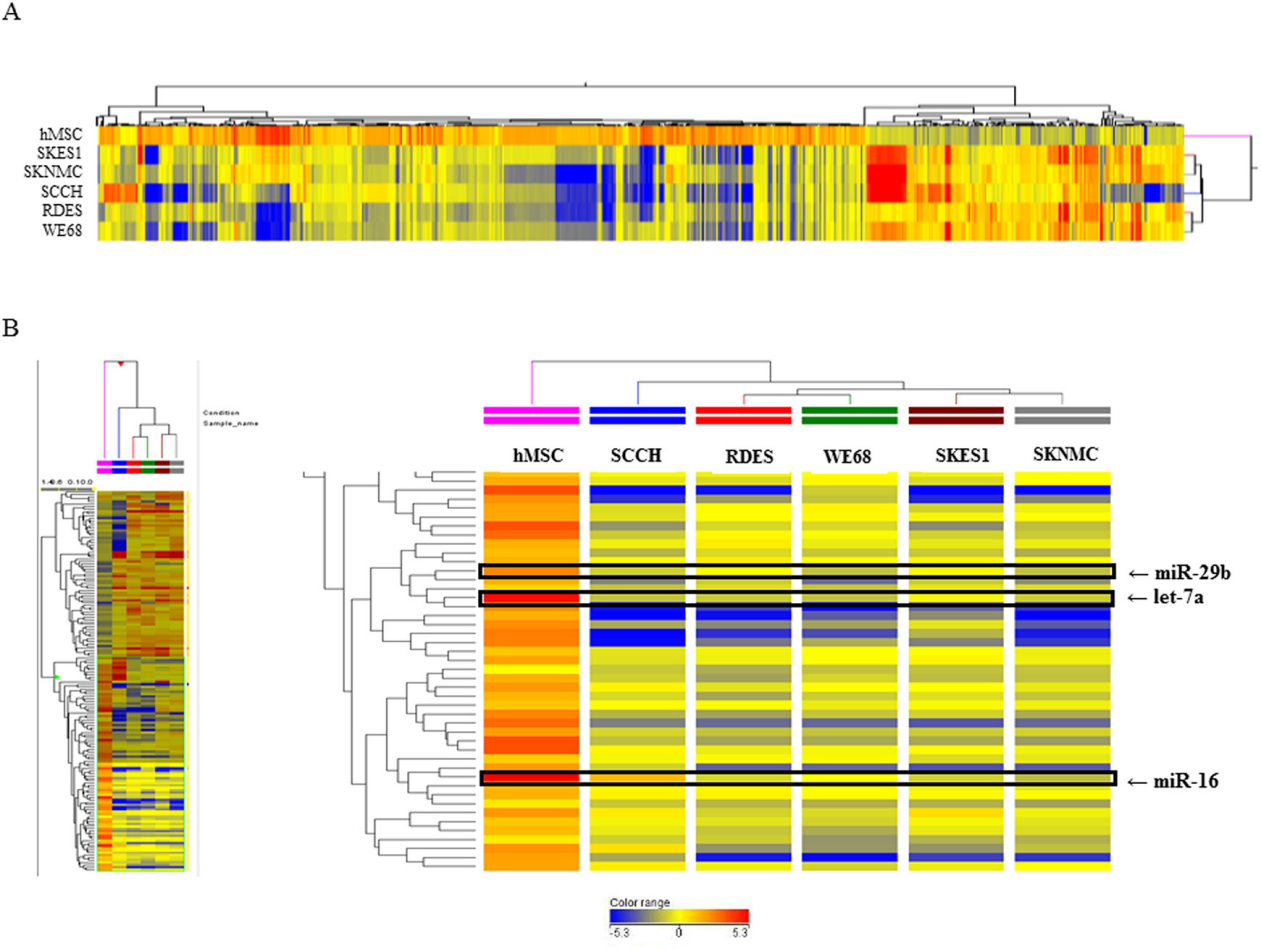
miRNA expression microarray. (A). Heat map of genome-wide miRNA profiles in five ES cell lines and hMSCs. (B), Let-7a, miR-16 and miR-29b are down regulated in all five OS cell lines. The color bar represents the grades of the relative expression levels; red color indicates an increase, while blue color indicates a decrease.

**Fig 2:**
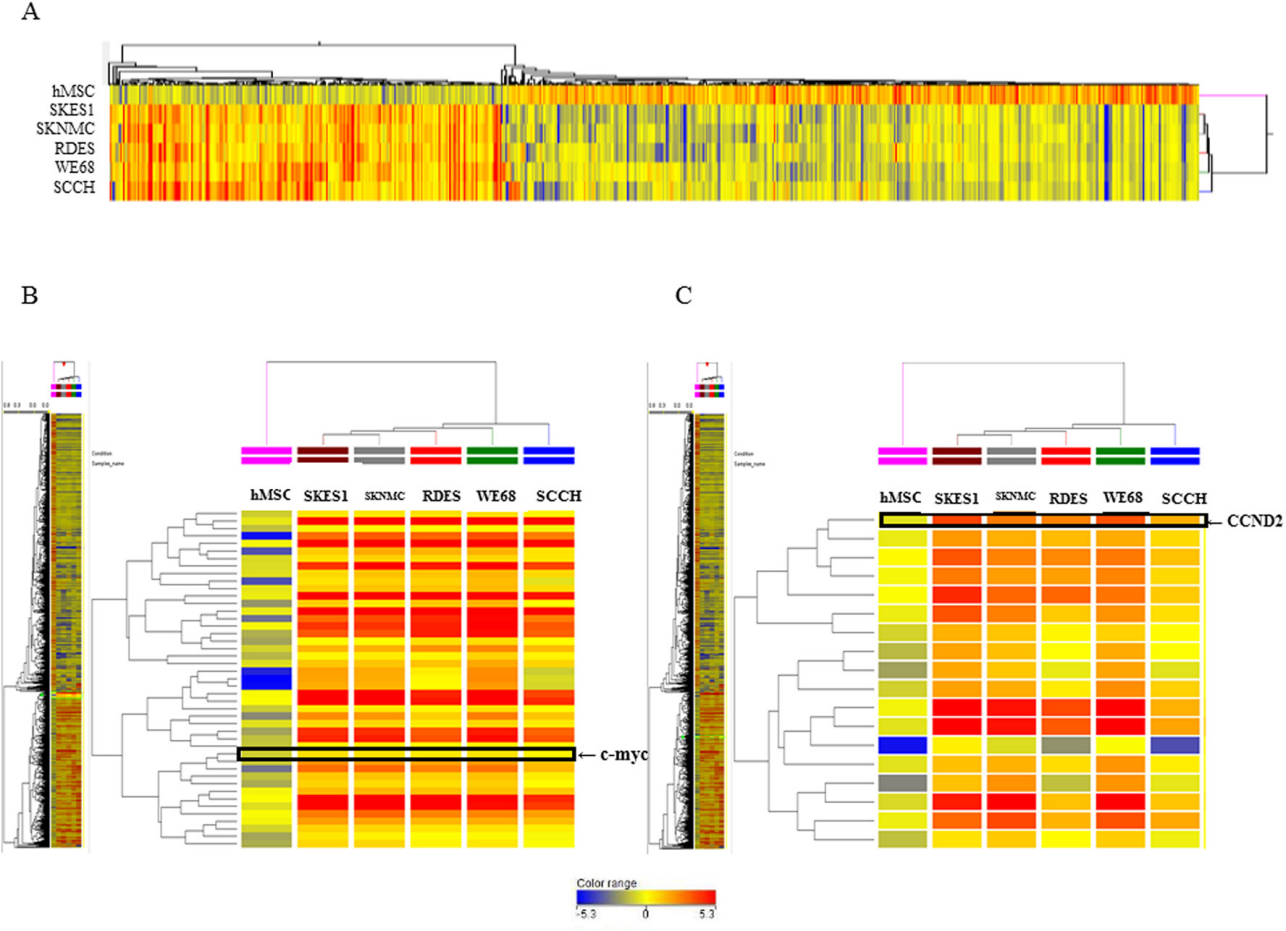
mRNA expression by cDNA arrays. (A), Whole-genome mRNA profile and extraction of c-Myc (B) and CNND2 (C), and the related genes followed the same pathways in ES cells and hMSCs. The color bar represents the grades of the relative expression levels; red color indicates an increase, while blue color indicates a decrease.

The genome-wide miRNA expression profiling of five ES cell lines was carried out to identify miRNAs specifically expressed in ES cells. The array analysis showed that the expressions of 1054 miRNAs in ES cells were significantly changed (fold-change >2.0) in comparison with hMSCs (Fig 1). Among 1054 miRNAs, 228 were significantly up-regulated, whereas 705 were significantly down-regulated in all tested ES cells compared to hMSCs. The remaining 121 miRNAs were reduced or increased among five ES cell lines. In the ES cell lines, the expression of let-7a was decreased by -24.15 ~ -46.15, miR-16 by -2.25 ~ -3.52, and miR-29b by -4.88 ~ -10.37 folds compared with hMSCs, respectively (Fig 2). In the SKES1, the expression of let-7a was decreased by -24.15, miR-16 by -2.81 and miR-29b by -5.2 folds compared with hMSCs, respectively.

### Up regulation of c-Myc and CCND2 expression in ES cell lines

**Fig 3:**
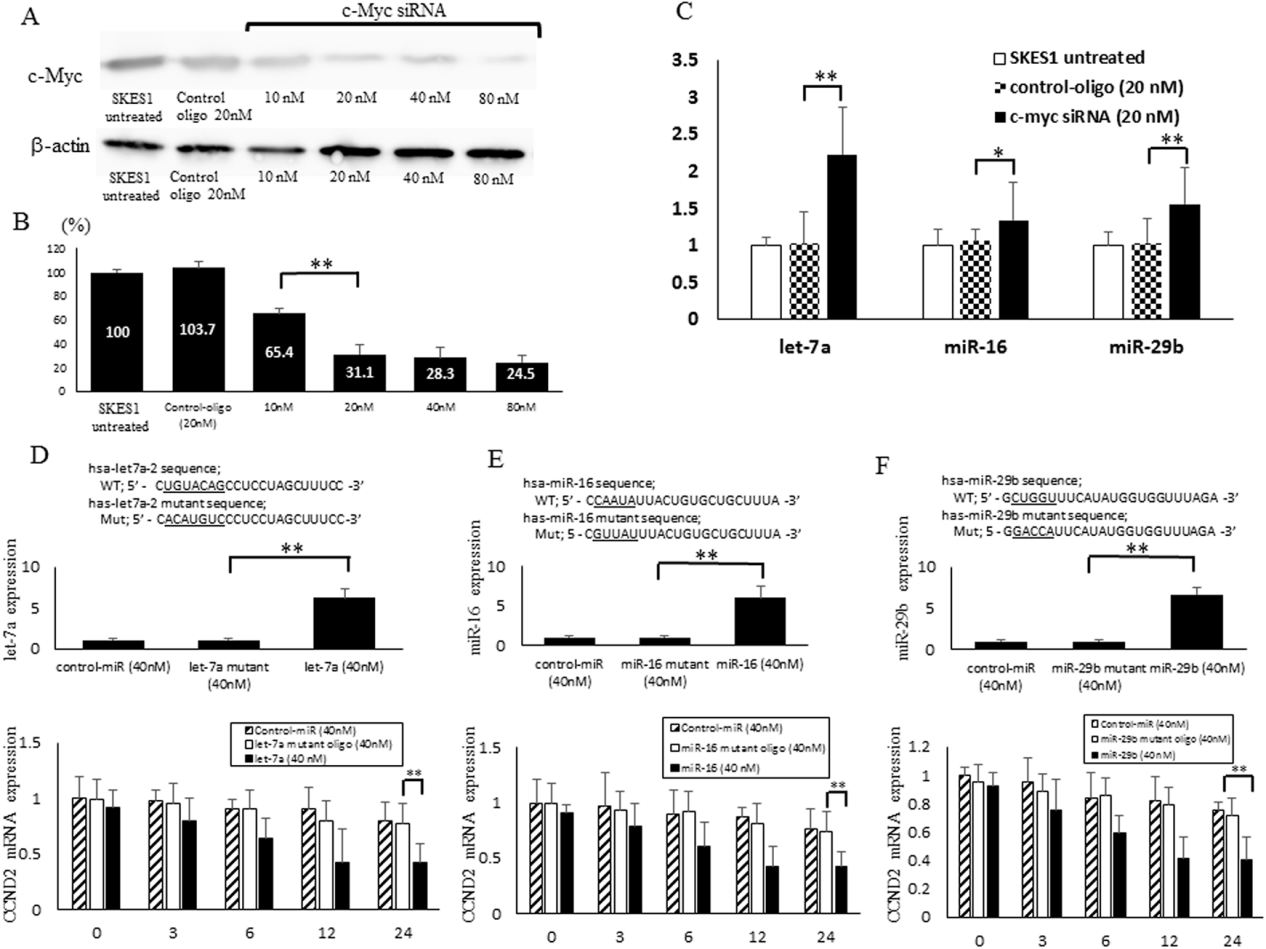
Silencing of c-Myc with c-Myc siRNA and let-7a, miR-16, miR-29b directly target to CCND2 mRNA in SKES1. (A), Transfection of c-Myc siRNA in SKES1 reduces the expression of c-Myc protein. (B), The quantification of c-Myc protein after transfection of c-Myc siRNA. (C), miRNA quantification of let-7a, miR-16 and miR-29b expression after transfection with c-Myc siRNA by quantitative RT-PCR. (D-F,) After actinomycinD treatment, the miRNA expression level of let-7a, miR-16 and miR-29b in the negative control-miR, let-7a, miR-16, miR-29b and their mutant was measured by qRT-PCR. After actinomycinD treatment, the mRNA expression level of CCND2 after transfection of negative control-miR, let-7a, miR-16, miR-29b and their mutant was measured by qRT-PCR. ANOVA was performed to statistically analyze the data. *p < 0.05 and **p < 0.01.

The cDNA array analysis demonstrated that the expressions of 3043 mRNAs were significantly changed (fold-change >2.0) between five ES cell lines and hMSCs (Fig 3A). We found that 1062 genes were significantly up-regulated, whereas 1884 genes were significantly down-regulated and the remaining 97 genes were up- or down-regulated in five ES cell lines compared to hMSCs. The expression of c-Myc was increased by 2.39 ~ 3.3 folds (Fig 3B.) and that of CCND2 was increased by 5.1 ~ 24.4 folds (Fig 3C) in five ES cell lines compared with hMSCs. In the SKES1, the expression of c-Myc was increased by 3.3 and CCND2 was increased by 24.4 folds compared with hMSCs respectively.

### Up-regulation of three tumor suppressive miRNAs after the knocking down of c-Myc

To study the roles of c-Myc in the regulation of let-7a, miR-16 and miR-29b in ES cells, we transfected the cells with siRNA targeting for c-Myc. The expression of c-Myc protein was down-regulated in c-Myc siRNA-transfected cells compared with untreated or negative control siRNA group (Fig 3A). The protein expression level of c-Myc in the cells transfected with c-Myc siRNA at 20 nM was reduced to 31.1% of that in the control cells (*p* < 0.01) (Fig 3B). On the contrary, the expression of let-7a (2.23 fold), miR-16 (1.35 fold), and miR-29b (1.56 fold) were significantly higher in c-Myc siRNA-transfected cells (20nM) compared with the untreated ES cells, as determined by real-time quantitative RT-PCR (Fig 3C).

### CCND2 as a direct let-7a, miR-16 and miR-29b target in ES Cell lines

The region complementary to the let-7a, miR-16 and miR-29b seed region was found in the 3’-UTR of human CCND2. A considerable complementarity between sequences within the seed regions of let-7a, miR-16 and miR-29b and sequences in the 3’-UTR of CCND2 was predicted, using the algorithms in BLAST and TargetScan. We blocked de novo mRNA transcription using actinomycin D (10 μg/ml), an inhibitor of mRNA transcription to determine changes in miRNA or mRNA stability. To test whether let-7a, miR-16 and miR-29b expression affected endogenous let-7a, miR-16 and miR-29b expression, we transfected let-7a, miR-16 and miR-29b mimic and their mutant oligonucleotides, as well as the negative control-miR, into SKES1 cells. We observed an increased let-7a, miR-16, miR-29b expression by 6.23 fold, 5.99 fold, 6.66 fold respectively compared with control-miR (Fig 3C.). Decreased CCND2 expression at the mRNA level following transfection with the let-7a, miR-16 and miR-29b mimic (Fig 3D–3F.).

### Inhibition of CCND2 expression by let-7a, miR-16 and miR-29b and CCND2-siRNA

**Fig 4:**
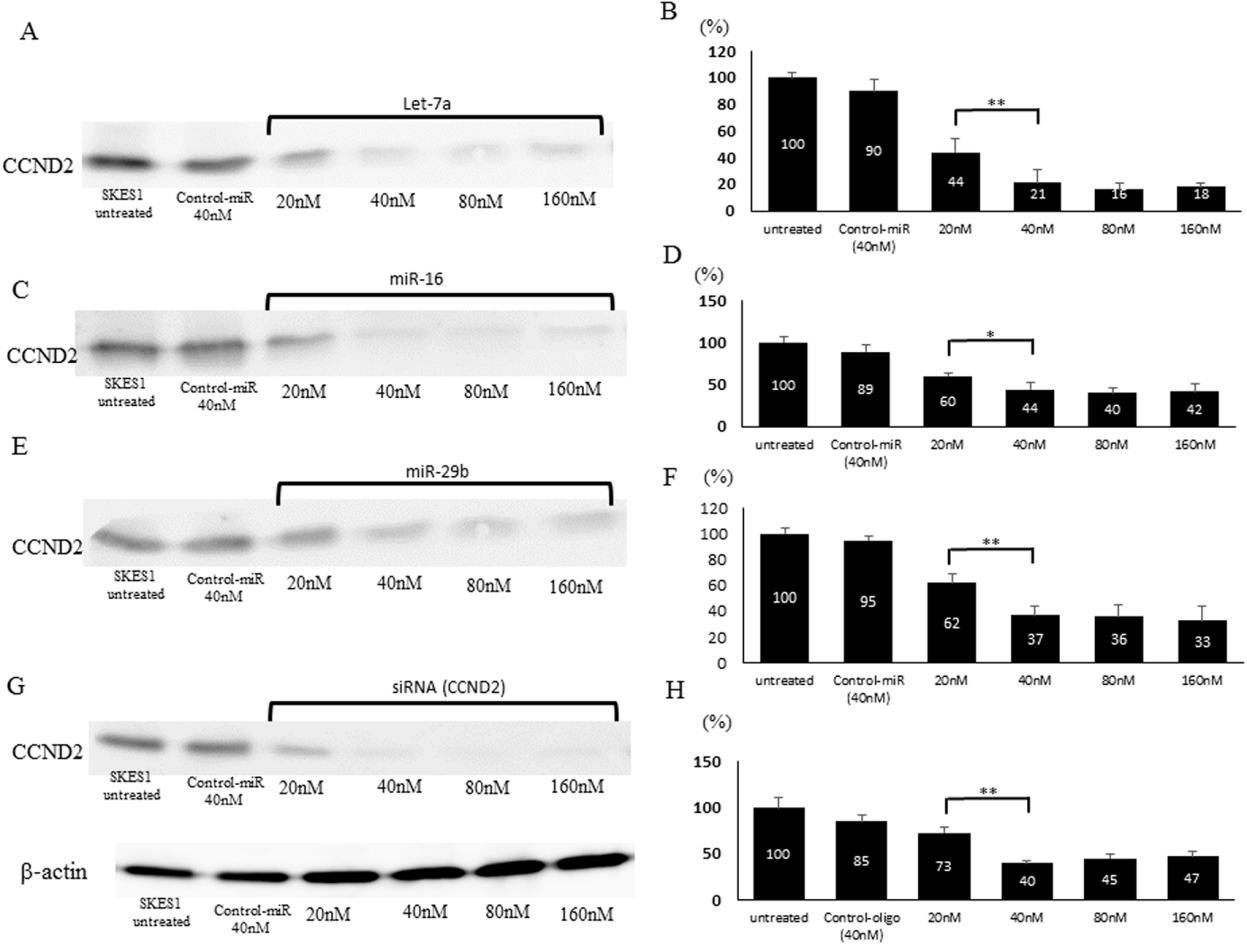
Silencing of CCND2 using let-7a, miR-16 and miR-29b and CCND2-siRNA in SKES1. The expression of CCND2 protein was decreased in SKES1 transfected with let-7a (A), miR-16 (C), miR-29b (E) and CCND2-siRNA (G). Densitometry quantification of CCND2 protein levels after transfection of let-7a (B), miR-16 (D), miR-29b (F) and CCND2-siRNA (H). *p < 0.05 and **p < 0.01.

To examine the correlation between let-7a, miR-16 and miR-29b and CCND2 in ES cells, these miRNAs were transfected into SKES1 cells. Western blot analysis showed that the expression levels of CCND2 dramatically decreased in all let-7a, miR-16, and miR-29b-transfected cells compared with negative control oligo-transfected cells (Fig 4A, 4C and 4E). The protein expression level of CCND2 in the let-7a, miR-16 and miR-29b-transfected cells (40nM) were reduced to 21.7%, 43.9% and 36.7% of that in the control cells, respectively (p < 0.05) (Fig 4B, 4D and 4F).

We also confirm the effects of CCND2-siRNA on the expression of CCND2 in ES cells. Although the expression level of CCND2 protein in the cells transfected with negative control siRNA was not significantly affected, that in the cells transfected with CCND2-siRNA was significantly reduced, as determined by Western blot (Fig 4G). Compared to the control cells (100%), CCND2 siRNA-transfected cells (40nM) exhibited the significantly lower CCND2 expression level by 40.2% (p < 0.01) (Fig 4H).

### Suppression of ES cell growth by transfection of let-7a, miR-16, miR-29b and CCND2-siRNA

**Fig 5:**
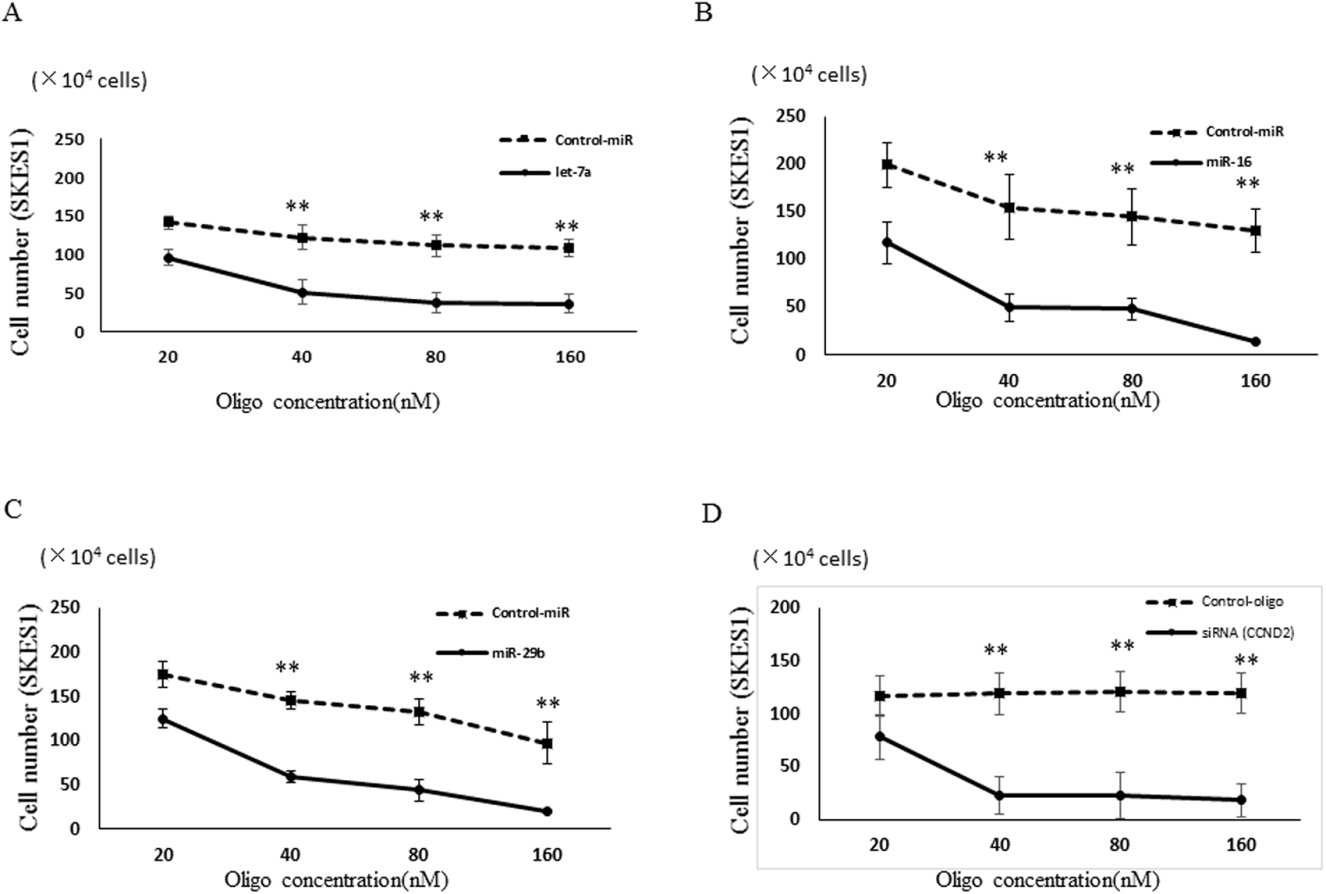
Cell proliferation assay. Antiproliferation effect of let-7a (A), miR-16 (B), miR-29b (C) and CCND2-siRNA (D) in ES cells. Error bars represent mean ± S.D. from three independent experiments. Two-tailed Student’s t-test was employed to statistically analyze the results. **p < 0.01

CCND2 is known to play important roles in the regulation of cell proliferation. Since the transfection of let-7a, miR-16, and miR-29b resulted in the reduction of CCND2 expression, we next examine the effects of let-7a, miR-16, and miR-29b on the proliferation of ES cells. The cell growth of SKES1 was inhibited by the transfection of let-7a, miR-16 and miR-29b in comparison with untreated and negative control miRNA transfected cells at 48 h after the transfection as determined by the cell counting (Fig 5A–5C). CCND2-siRNA transfected SKES1 cells, as same as let-7a, miR-16 and miR-29b transfected cells, showed significant inhibition of the cell proliferation compared with the negative control siRNA transfected cells (Fig 5D). Consistent with the above data, the results demonstrated that CCND2 was the down-stream effector of let-7a, miR-16 and miR-29b, which were regulated by c-Myc.

### Inhibition of cell cycle progression at G0/G1 phase by let-7a, miR-16 and miR-29b

**Fig 6:**
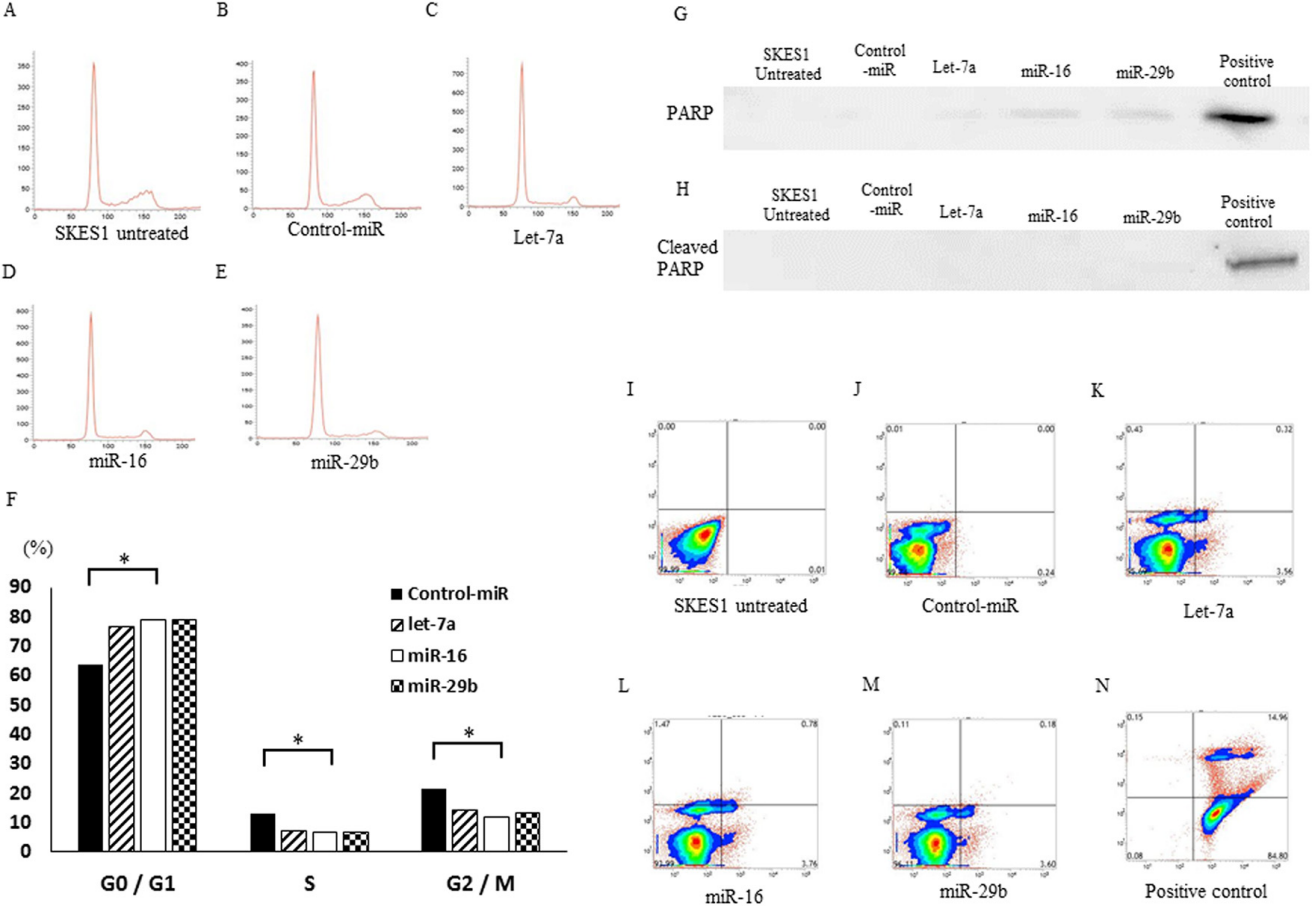
Effects of let-7a, miR-16 and miR-29b miRNAs on the cell cycle in SKES1. The SKES1 were treated and analyzed by flow cytometry after PI staining (A–E). Histogram shows the quantitative percentage of diploid cells (DNA content) in each cell-cycle phase (F). Effects of let-7a, miR-16 and miR-29b on the induction of apoptosis in SKES1 cells. Western blotting shows the expression of PARP (G) and its cleaved form (H). The cells were labeled with FITC annexin V and PI (I–N). Each quadrants represent viable cells (Lower Left quadrant), early apoptotic cells (Lower Right), late or secondary necrotic cells (Upper Right), and primary necrotic cells (Upper Left), respectively. *p < 0.05.

Since the introduction of let-7a, miR-16 and miR-29b significantly inhibited cell proliferation of ES cell lines, we hypothesized that these miRNAs might induce the cell cycle retardation and⁄or apoptosis of the cells. To monitor the cell cycle distributions, FACS analyses were carried out after the miRNAs transfectiton (Fig 6C–6E). In the miRNAs transfected cells, the number of cells in G2⁄M and G0/G1 phase was significantly lower and higher than that in the untreated (Fig 6A) or control oligo transfected cells (Fig 6B), respectively (Fig 6F). The data demonstrated that the restoration of let-7a miR-16 and miR-29b resulted in the cell cycle retardation at G0⁄G1 phase in ES cells. Then, the cellular expression of PARP and its cleaved product was assayed by immunoblotting in SKES1 cells and their transfectants (Fig 6G). The cleavage of PARP protein, a marker of caspase-mediated apoptosis, was not observed in miRNAs (let-7a, miR-16 and miR-29b) transfectants as well as untreated cells and negative control transfectants, in marked contrast to ADM-treated (positive control) cells (Fig 6H). Furthermore, in flow cytometry analysis using Annexin V-FITC/PI double staining, there were no significantly differences in the distribution patterns between untreated (Fig 6I), negative control miRNA (Fig 6J), let-7a (Fig 6K), miR-16 (Fig 6L) or miR-29b (Fig 6M) transfected cells compared to the positive control cells exhibiting apoptosis (Fig 6N). The programmed cell death was not induced by let-7a, miR-16 or miR-29b in ES cells.

### Inhibition of cell cycle progression by transfection of siRNAs for c-Myc and CCND2 in ES cells

**Fig 7:**
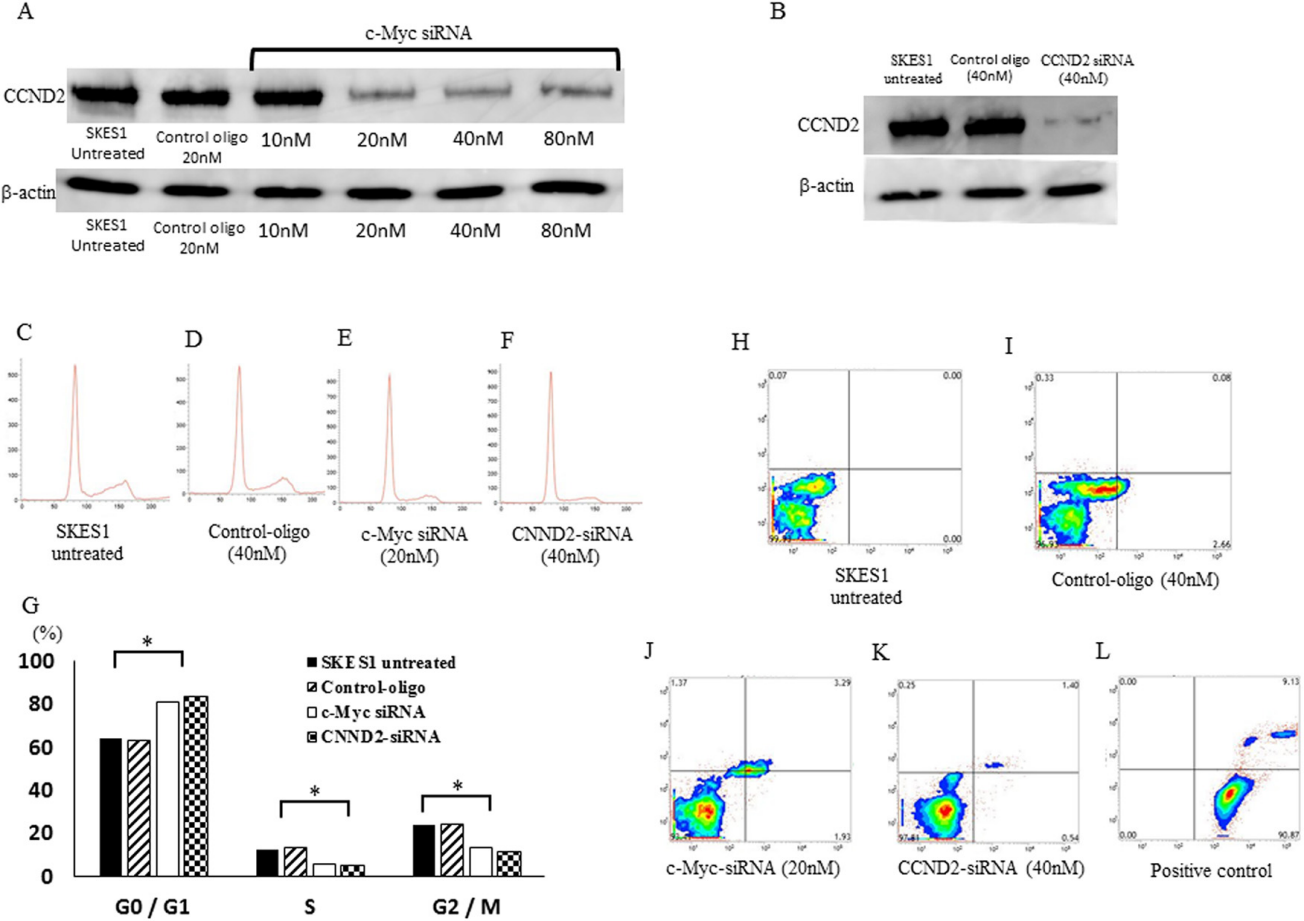
Effects of c-Myc and CCND2-siRNA on the cell cycle and apoptosis in SKES1. (A), Transfection of c-Myc siRNA in SKES1 reduced the expression of CCND2 protein. (B), Transfection of CCND2 siRNA in ES cells reduced the expression of CCND2 protein. The cells were treated and analyzed by flow cytometry after PI staining (C–F). Histogram shows the quantitative percentage of diploid cells (DNA content) in each cell-cycle phase in c-Myc and CCND2-siRNA treated cells (G). Effects of c-Myc and CCND2-siRNA on induction of apoptosis in the SKES1 cells. The cells were labeled with FITC annexin V and PI (H–L). *p < 0.05.

To verify the correlation of c-Myc and CCND2 in regulating ES cell activities, we transfected the cells with siRNA targeting c-Myc. The expression of CCND2 after the transfection with c-Myc siRNA was significantly lower than that in non-treated and negative control siRNA transfected cells as determined by Western blot (Fig 7A). CCND2-knockdown cells also showed repressed expression of CCND2 (Fig 7B). In addition, CCND2 siRNA transfection inhibited the cell proliferation and cell cycle progression in ES cells. These results suggested that CCND2 is the down-stream effector of c-Myc, and is one of the important factors for tumorgenesis in ES cells. Both in c-Myc (Fig 7E) and CCND2-siRNA (Fig 7F) transfected cell lines, the number of the cells in G2⁄M and G0/G1 phases was significantly lower and higher than that in the untreated (Fig 7C) or negative-control siRNA (Fig 7D) transfected cells, respectively (Fig 7G). The flow cytometry analysis using Annexin V-FITC/PI double staining, there were no significantly differences in the distribution patterns between untreated (Fig 7H), negative control oligo (Fig 7I), c-Myc siRNA (Fig 7J) or CCND2 siRNA (Fig 7K) transfected cells with contrast to the positive control cells exhibiting apoptosis (Fig 7L). The data were consistent with the above results of miRNA transfection experiments. These observations suggested that knockdown of c-Myc might induce CCND2 repression resulting in the cell cycle retardation at G0⁄G1 phase, but not induce apoptosis in ES cells.

### Inhibition of tumor growth in nude mice xenograft model by let-7a, miR-16 and miR-29b

**Fig 8:**
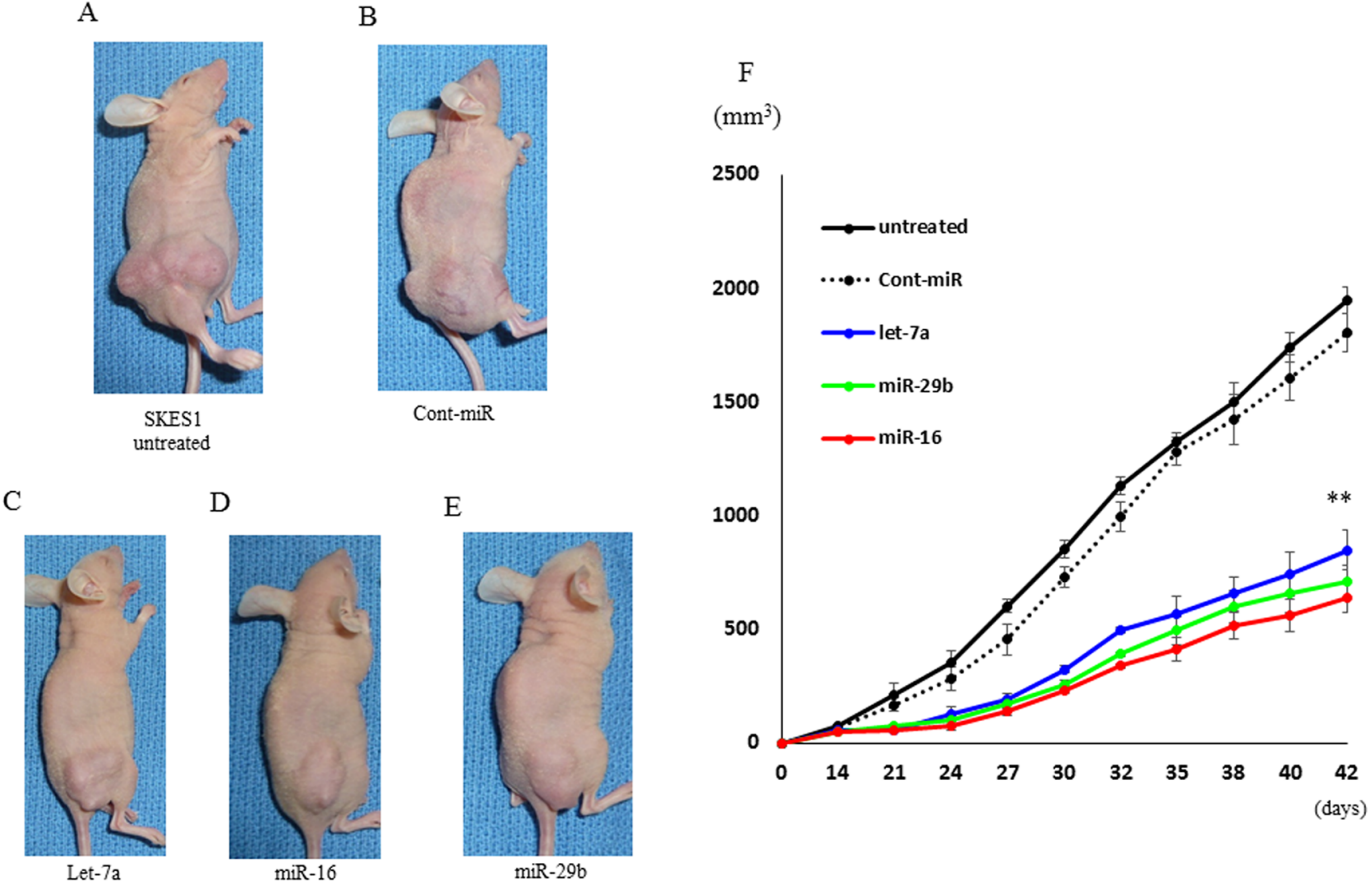
Let-7a, miR-16 and miR-29b suppressed the ex vivo treatment tumor growth. The five groups included (A) untreated (n = 5), (B) transfected with negative control miRNA (n = 5), and (C) transfected with let-7a (n = 5), (D) transfected with miR-16 (n = 5) and (E) transfected with miR-29b (n = 5). Tumor volumes were measured at the indicated time points after tumor cell inoculation. **p < 0.01.

We next investigated the efficacy of let-7a, miR-16 and miR-29b on the tumor growth ex vivo treatment (S1 File). The introduction of let-7a, miR-16 and miR-29b miRNAs into SKES1 cells resulted in the decreased growth of subcutaneous xenografted tumors in nude mice (Fig 8A–8E). SKES1 cells transfected with the miRNAs showed statistically smaller tumors in mice compared to untreated (1949.2 ± 57.9 mm^3^) and negative control miRNA transfected groups (1805 ± 83.9 mm^3^) (Fig 8F), indicating that let-7a (848 ± 85.1 mm^3^), miR-16 (636.8 ± 64.2 mm^3^) and miR-29b (711.8 ± 71.6 mm^3^) inhibited the growth of ES cells ex vivo treatment.

## Discussion

During the last decade, over 700 human miRNAs have been identified and a massive evidences has been identified to demonstrate the important roles of miRNAs in oncogenesis such as differentiation, proliferation and apoptosis [19]. Aberrant expression of miRNAs in human cancer cells causes destruction of miRNA-mediated mRNA networks. Featured miRNAs have been shown to act as critical factors for tumorigenesis or tumor suppressors emphasizing the importance of identification of miRNAs that function in the regulation of tumor progression. Among the various families of miRNAs, the let-7a, miR-16 and miR-29b have become the prototypes for miRNAs that function as the tumor suppressors since these miRNAs could inhibit the expression of multiple oncogenes, including c-Myc [20–22]. To identify important factors involved in miRNAs-mRNAs network in ES, we performed genome-wide miRNA array as well as cDNA array in the same ES cells.

In the present study, miRNA array results demonstrated that the expression of let-7a, miR-16 and miR-29b were down-regulated in all of five ES cell lines. Several studies have shown that let-7a, miR-16 and miR-29b are down-regulated and are closely related to the abnormal potentials in malignant tumors [23–25]. However, the biological roles of let-7a, miR-16 and miR-29b in ES cells have not been clarified yet. The results indicated that the expression of let-7a, miR-16 and miR-29b was coordinately up-regulated in ES cell lines, and made us to investigate genome-wide mRNA profiling by cDNA array to detect the possible targets of let-7a, miR-16 and miR-29b in ES cells.

The data from cDNA array analyses showed that c-Myc and CCND2 mRNA expression was commonly increased in five ES cell lines compared with hMSCs. Furthermore, the sequence analysis suggested possible association of let-7a, miR-16 and miR-29b with 3’UTR of CCND2 (S1 Fig). c-Myc has strong ability to promote cell cycle progression [26], and aberrant expression of c-Myc leads to abnormal cellular proliferation. CCND2 is a member of cyclin family of transcription factors, and has been well-identified as an important regulator of cell cycle [27]. Up-regulation of c-Myc and CCND2 has been found in several types of cancer and our data is consistent with the previous studies reporting that the up-regulation of c-Myc and CCND2 may contribute to malignant potentials of ES.

The significant suppression of let-7a, miR-16 and miR-29b expression in ES cells suggests the tumor suppressive roles of these miRNAs in ES. It is reported that these miRNAs function as tumor suppressors and their expressions are regulated by c-Myc [28–30]. Although let-7a, miR-16 and miR-29b might influence the expression of several genes, we focused on CCND2 as the target of the miRNAs in ES cells. Our cDNA array analysis demonstrated that CCND2 was uniformly up-regulated in all five ES cell lines, whereas the expression of other candidates of target genes differed among ES cells. The analysis using several algorithms, such as BLAST, and real-time PCR after miRNAs transfection, further suggested that CCND2 was directly targeted by let-7a, miR-16 and miR-29b. It is possible that let-7a, miR-16 miR-29b may have down-regulated CCND2 expression via indirect pathway. However, CCND2 mRNA degradation was promoted even after the termination of de novo mRNA transcription. The effect was not observed in the mutant miR-treated cells. Therefore, let-7a, miR-16 miR-29b may have affected CCND2 mRNA directly at least in part. Thus, the expression analysis data lead us to the prediction of new axis that c-Myc might repress the tumor suppressive miRNAs, let-7a, miR-16 and miR-29b, and the inhibition of these miRNAs might result in the up-regulation of CCND2 in ES cells.

Our data suggests that c-Myc might negatively regulate let-7a, miR-16 and miR-29b expression in ES cells. c-Myc encodes basic helix-loop-helix zipper transcription factor which is usually dysregulated in cancer, and exhibits the advantageous effects on cancer cell growth, proliferation, survival and metastasis [31,32]. It has been reported that Myc-repressed miRNAs (MRMs) are validated as direct targets of c-Myc, and that MRMs are negatively regulated in cancers [33–35]. In the present study, knockdown of c-Myc using siRNA revealed the up-regulation of let-7a, miR-16 and miR-29b, indicating that these tumor suppressive miRNAs are also regulated by c-Myc as MRMs in ES cell lines.

We next examined the functions of let-7a, miR-16 and miR-29b in the regulation of their possible target gene, CCND2, and the changes in the biological characteristics in ES cell lines. The present study demonstrated that forced elevation of let-7a, miR-16 and miR-29b resulted in the reduction of the expression of CCND2 protein in ES cells. Dong, et al reported that CCND2 is the direct target of let-7a in prostate cancer [15]. It is also shown that CCND2 is directly regulated by so-called tumor suppressive miRNAs [36, 37]. Our results suggested that the same regulatory mechanism of CCND2 expression via let-7a, miR-16 and miR-29b might exist in ES cells.

Our data regarding the cell cycle analysis showed that let-7a, miR-16 and miR-29b inhibited the proliferation of ES cells via cell cycle retardation at G1/G0 phase. These observations are consistent with the previous report demonstrating that CCND2 is necessary to bypass the normal G1/S checkpoint [38]. It is noteworthy that the down-regulation of CCND2 by challenge of let-7a, miR-16, miR-29b or siRNA against CCND2 did not induce apoptosis in ES cells, indicating that the repression of ES cell growth was acquired by the cell cycle retardation.

We also explored whether c-Myc would regulate the expression of let-7a, miR-16 and miR-29b and these tumor suppressive miRNAs suppress the expression CCND2. So that means the down-regulation of c-Myc should induce the repression CCND2 and cell cycle retardation. Indeed, debilitation of c-Myc using siRNA revealed down-regulation of CCND2, and induced the correction of abnormal cell cycle progression, which were consistent with the results of the transfection experiments with let-7a, miR-16 and miR-29b in ES cells.

Furthermore, ex vivo treatment studies showed the inhibition of ES tumor cell growth in mice injected with ES cells overexpressing of let-7a, miR-16 or miR-29b. As consistent with the data of in vitro experiments, the xenograft model of ES also indicated that let-7a, miR-16 and miR-29b induction had the ability to inhibit ES cells development ex vivo treatment by targeting CCND2 expression.

In summary, the present study showed that the down-regulation of let-7a, miR-16 and miR-29b were mediated by c-Myc and subsequently inhibited the expression of CCND2 in SKES1. c-Myc is called as “Maestro of microRNAs” [39], and is essential for many important tumor phenotypes including cell proliferation and tumor growth. c-Myc exhibited inverse correlation with let-7a, miR-16 and miR-29b, and these tumor suppressive miRNAs played important roles in SKES1 proliferation and tumorigenesis by targeting CCND2 both in vitro and ex vivo treatment. CCND2 expression is shown to increase as progression of stages of malignant tumors, with the highest activities was observed in metastatic lesions [40]. Our data also suggest that CCND2 is one of the crucial factors to enhance tumor proliferation in ES, as other malignant tumors. Although the confirmation of the data demonstrated in the present study using clinical samples of ES should be required in the future, the novel information presented here regarding the hideous axis in ES cells would be beneficial for the better understanding of biology of ES and might provide a certain aspect of therapeutic application for ES.

## Supporting Information

S1 Fig Target Prediction of miRNAs. Predicted binding sites of let-7a (A), miR-16 (B), and miR-29b (C) in 3′-UTR of CCND2, as aligned by Basic Local Alignment Search Tool (BLAST), TargetScan 6.0 (microRNA.org).
(TIF)

S1 File Individual tumor size. The experimental tumor bearing mice model was established and tumor volumes were measured for 6weeks after tumor inoculation.
(TIF)
