# Supplementary figures and images for: c-Myc Represses Tumor-Suppressive microRNAs, let-7a, miR-16 and miR-29b, and Induces Cyclin D2-Mediated Cell Proliferation in Ewing’s Sarcoma Cell Line
Source: PLoS One. 2015 Sep 22;10(9):e0138560. doi: 10.1371/journal.pone.0138560 (PMC4578885; doi:10.1371/journal.pone.0138560)

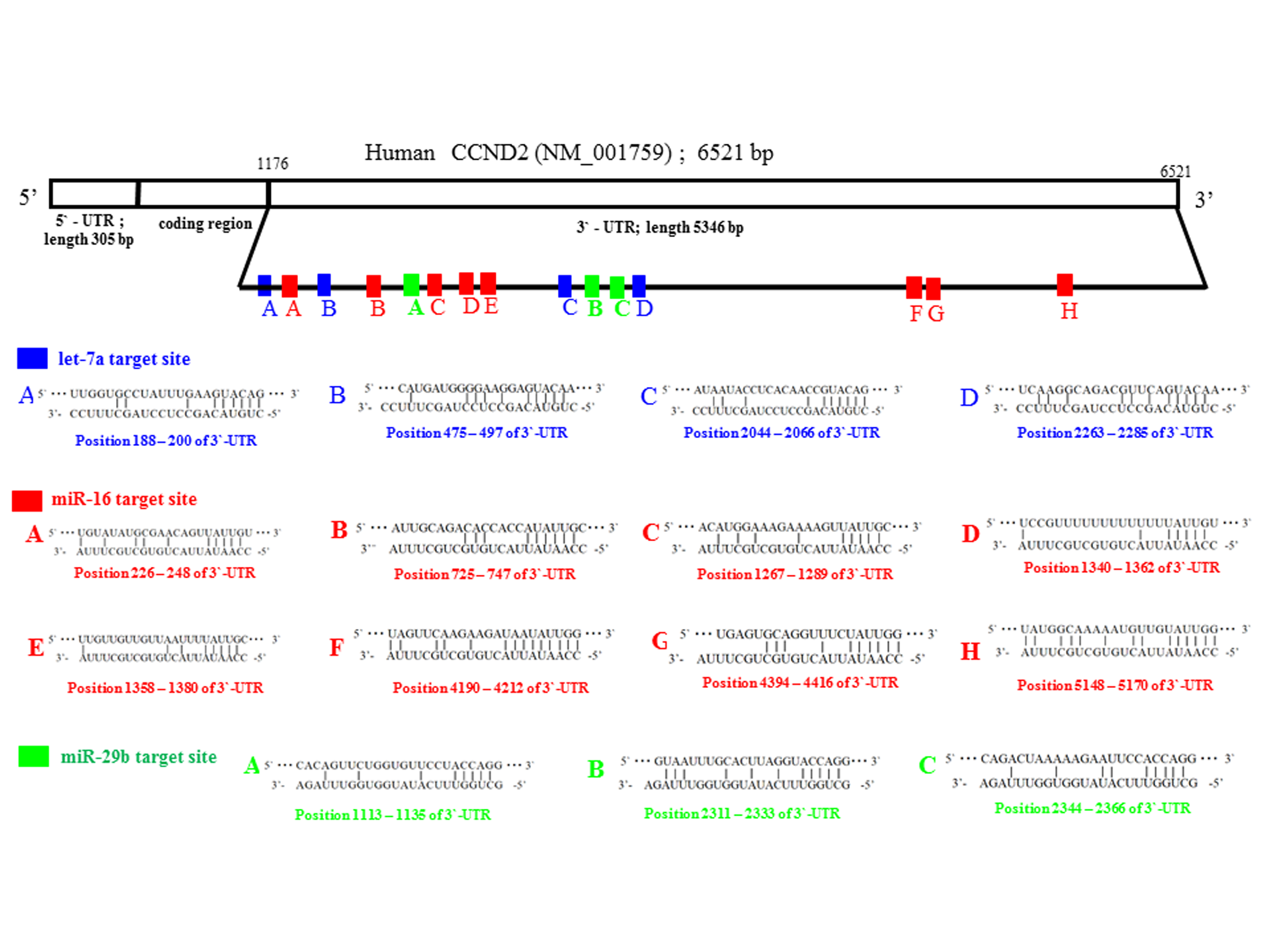

Supplement: S1 Fig — Predicted binding sites of let-7a (A), miR-16 (B), and miR-29b (C) in 3′-UTR of CCND2, as aligned by Basic Local Alignment Search Tool (BLAST), TargetScan 6.0 (microRNA.org). (TIF) [file pone.0138560.s001.tif]

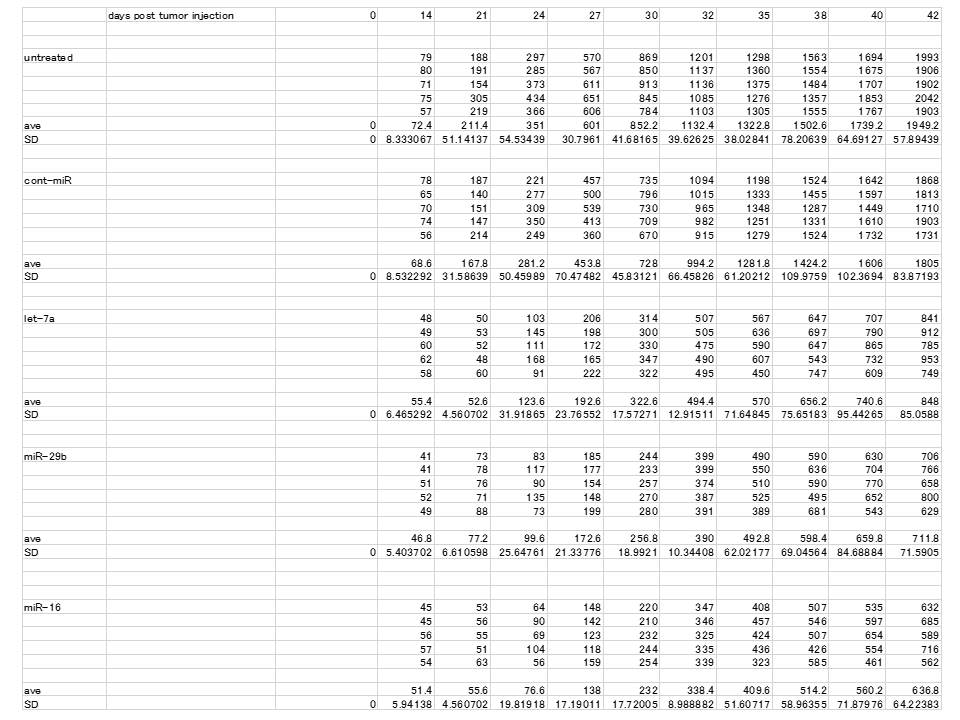

Supplement: S1 File — The experimental tumor bearing mice model was established and tumor volumes were measured for 6weeks after tumor inoculation. (TIF) [file pone.0138560.s002.tif]
